# Renal Adenomas: Pathological Differential Diagnosis with Malignant Tumors

**DOI:** 10.1155/2008/974848

**Published:** 2008-10-08

**Authors:** F. Algaba

**Affiliations:** Pathology section, Fundació Puigvert, Universitat Autónoma de Barcelona (UAB), 08025 Barcelona, Spain

## Abstract

The renal adenomas can be confused by imaging diagnosis with malignant renal tumors, but there are also real biological dilemmas to determine their behavior. The consensus decisions are the following. (1) The adenoma of clear cells is not accepted, instead it is considered that all the clear-cell tumors are carcinomas, with greater or lesser aggressiveness. (2) Among the papillary neoplasms the WHO 2004 renal cell tumors classification are considered as papillary adenomas tumors with a maximum diameter of 5 mm and may represent a continuum biological process to papillary renal cell carcinoma. The papillary adenomas associated with End-kidney and/or acquired cystic disease may have a different pathogenesis. (3) To consider a tumor as an oncocytoma the size is not important, only the cytological features, microscopic, ultrastructural, and immunohistochemically can help, but some chromosomal observations introduce some questions about its relation with the chromophobe renal cell carcinoma. (4) Finally, the metanephric adenoma, a tumor with some morphological similarity with the nephroblastoma must be considered in the renal adenomas diagnosis.

## 1. INTRODUCTION

Before sonographic studies, 85% to 90% of renal masses were malignant, with the majority being renal-cell carcinoma. However, with the increasing frequency of incidentally discovered renal masses, only 70% to 85% of lesions are found to be malignant [1].

When we take on the subject of benign neoplasias of the kidney, we must make two large groups, the benign mesenchymal neoplasias and the benign epithelial neoplasias or adenomas.

The benign mesenchymal tumors, with the exception of the angiomyolypoma, are usually subclinical and rarely give the pathologist diagnostic problems, although they can be confused with malignant neoplasias for imaging diagnosis [2].

The adenomas are a true clinical-pathological dilemma, not only because they can be confused by imaging diagnosis, but because there are biological dilemmas to determine and therefore different questions emerge: firstly, do the renal adenomas really exist?, and secondly, in case they do exist, are they precursor lesions of renal carcinomas?, and if they were, do we have the possibility of differentiating the benign neoplasias from the malignant ones?

The current classifications of renal carcinomas have managed to integrate the genetic and molecular findings with the cytological characteristics [3]. This conjunction has made it possible to correlate the histological subtypes with the prognostic and therapeutic ones. For this reason, we can approach the renal adenomas according they are of clear cells, of eosinophilic cells (oncocytes), with papillary growth, or have a metanephric blastema appearance.

## 2. RESULTS

### 2.1. Adenomas with clear cells?

The most frequent renal neoplasia of the adult is the clear cell renal cell carcinoma. When the pieces from the nephrectomy are studied with this type of carcinoma, around 10% of the cases are multifocal and small tumor nodes with clear cells can be found. This same finding can be made more frequently in kidneys of patients with von Hipel-Lindau disease. These nodes could have been considered adenomas of clear cells, but since Bell's descriptions [4] it is well known that some of the small clear-cell tumors have metastasis capacity and therefore currently the existence of an adenoma of clear cells is not accepted, instead it is considered that all the clear-cell tumors are carcinomas, with greater or lesser aggressiveness.

**Figure 1:**
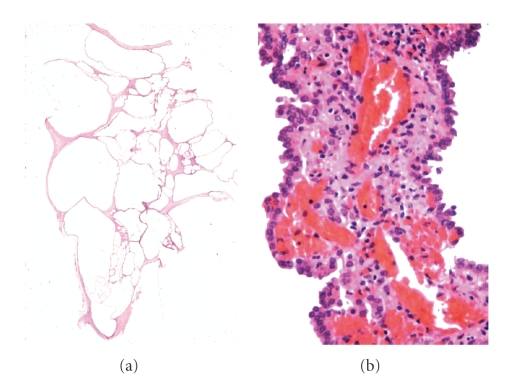
Cystic nephroma: cystic neoplasm with fibrous stroma an flat epithelium covering the wall.

Having established this axiomatic attitude, there is no problem of differential diagnosis; however, from the morphological point of view, the *cystic nephroma* (Figure 1), formed by multiple separate cysts (which are also known as multilocular cyst) covered by epithelium without nuclear atypia, monolayer, with eosinophilic cytoplasma, can occasionally be covered by cells of clear cytoplasma, without nuclear atypia. In this case, clear cells must not be found in the walls and the intercystic stroma. The cystic nephroma does not have any relation to the multilocular clear cell carcinoma (despite certain similarity with it) [5]. Currently, it is being related to other benign neoplasias such as the mixed epithelial and stromal tumor of the kidney, all of them are much more frequent in women and with estrogen and progesterone receptors in the stromal component [6].

### 2.2. Papillary adenomas

In about 35% of the cases the renal carcinomas with a papillary pattern have multiple lesions of diverse sizes (from millimeters to centimeters), especially those associated with family syndromes. This fact again poses the existence of adenomas and their possible relation with carcinomas.

**Figure 2:**
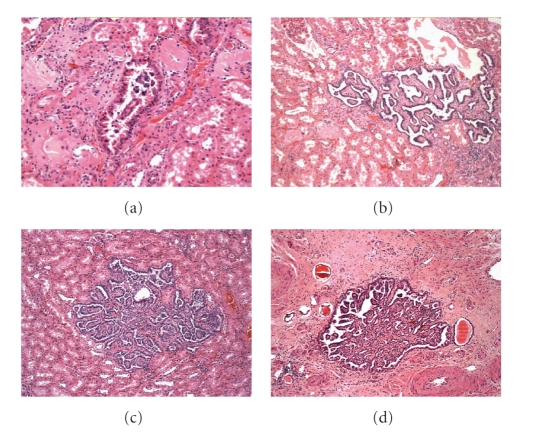
Papillary adenomas: different types of basophilic cell adenomas.

The small papillary tumors are characterized by a growth of cells with scant cytoplasma (chromophilic cells), occasionally somewhat eosinophilic, with tubular-papillary patterns, well delimited and not encapsulated (Figure 2). In chromosomal studies, trisomies in chromosomes 7 and 17 were confirmed in the small tumors. Additionally, other chromosomes presented tri-tetrasomies when the in size of the tumor increase. From these findings it was considered that there is a series of small benign lesions and that the increase in size is associated with greater amount of chromosomal alterations and therefore the possible transformation in papillary carcinomas. For this reason, the WHO 2004 renal cell tumors classification considered tumors with a maximum diameter of 5 mm as papillary adenomas [3]. In a practical manner, many pathologists consider that the tumors over 5 mm and up to 10 mm are of low aggressiveness [7].

It should be underscored that although the majority of the papillary adenomas are associated with papillary renal cell carcinoma (47%), they can also be found associated with other variants (16% with clear cell RCC, 8% with chromophobe RCC and 2.5% with oncocytoma) [8].

It should be highlighted that 5% of the papillary adenomas are found in sclerosed kidneys (end-kidneys) and 18% in patients with acquired cystic disease (with or without dialysis). Their morphological characteristics are identical to those associated with carcinomas but curiously they differ from the latter by not expressing alpha-methylacyl-CoA racemase (AMACR) [8].

In conclusion, papillary adenoma and papillary renal cell carcinoma may represent a continuum of the same biological process. Unfortunately, it is not possible to define an unequivocally benign papillary renal adenoma, for this reason the WHO used the size (arbitrarily) as a marker.

The papillary adenomas associated with end-kidney and/or acquired cystic disease may have a different pathogenesis.

### 2.3. Oncocytoma

The clinically most important renal cortical adenoma is the oncocytoma, since despite the fact that it is not usually associated with the carcinoma, in the imaging diagnosis it is usually considered as renal cell carcinoma.

The cytological characteristics of the oncocytoma are defined by the oncocytic cells (tumor cells arranged in nests, cords, or tubules, with eosinophilic cytoplasm and no mitosis). They are usually solid, homogeneous, with occasional sclerosed central areas, which can also present in other tumors, and are of a diameter from millimeters up to 12–15 or more centimeters. Therefore, in this tumor type, the criterion of size does not exist [9].

**Figure 3:**
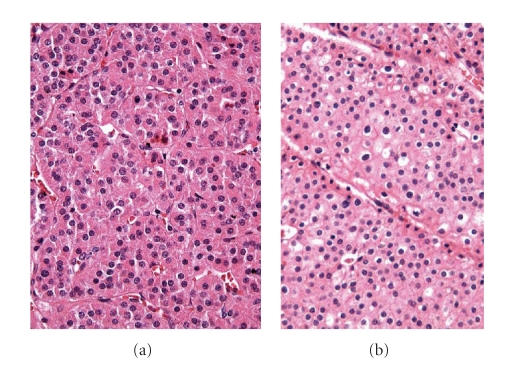
(a) Oncocitomas, (b) eosinophilic chromophobe renal cell carcinoma variant. Notice the similar aspect that both lesions can present.

The problem originates in the cytological characteristics that at times are difficult to distinguish from other neoplasias of eosinophilic cells, such as the clear-cell renal carcinomas eosinophilic variant and especially the chromophobe eosinophilic carcinomas (Figure 3).

**Figure 4:**
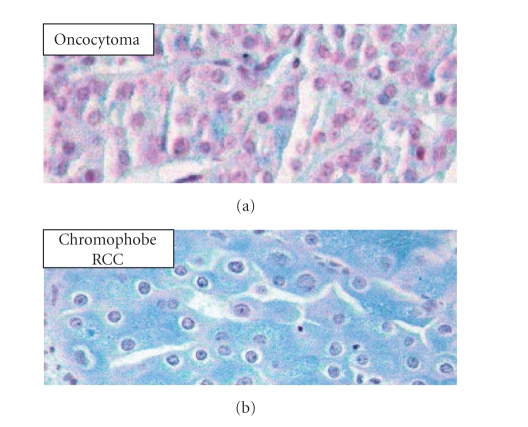
Colloidal iron to distinguish oncocytoma (negative) and chromophobe renal cell carcinoma (positive).

To distinguish them, the electronic microscope, histochemistry (colloidal iron), and immunohistochemistry can help (Figure 4).

It is interesting to point out that the chromosomal studies have demonstrated different types of alterations, and therefore while some tumors do not have any chromosomal alteration, others show translocation 11q13 and (−) 1p, 14q, Y [10]. The latter chromosomal alteration is similar to that of the chromophobe carcinomas (−1p, Y), which together with the finding of hybrid carcinomas (oncocytoma + chromophobe renal cell carcinoma) especially in the Birt-Hogg-Dubé syndrome [11] it has suggested that certain cases of oncocytomas could evolve into chromophobe renal cell carcinoma.

### 2.4. Metanephric adenoma

**Figure 5:**
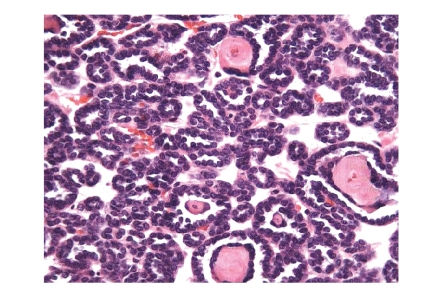
Metanephric adenoma. microacinar structures of basophilic cells with a nephroblastic appearance.

A relatively short time ago a tumor was introduced among the renal adenomas that was comprised by small cells with scant cytoplasma, uniform, without mitosis, embryonic-appearing, distributed in small round acini with a phenotype similar to the nephroblastoma (Figure 5). They represent 1% of localized tumors of less than 7 cm. The mean age is 41 years (from 5 to 83 years). Fifty percent are incidental and 10% have a polycythemia. Immunohistochemistry, the WT1, CD 56, and CD 57 are positive and the AMACR is negative [12].

From the genetic point of view, it is characterized by allelic loss in 2p13 (56% of the cases) and is differentiated from the nephroblastoma (with alterations 11p13) and from the papillary carcinoma (+7, +17) [13].

To distinguish the metanephric adenoma from the nephroblastoma, strict diagnostic criteria have been used, and only accepting the tumors without mitosis and nucleoli as metanephric adenoma.

## 3. CONCLUSIONS

We see that the criteria used to consider renal neoplasia as adenoma vary a great deal according to the cellular type (never in the neoformations of clear cells, only in small neoplasias of papillary pattern, and any size if we are sure that they are oncocytic or metanephric cells). Therefore, it is fundamental to establish the cellular type, and this determination is usually done with the usual pathological anatomical methods with the help of the immunohistochemical markers to which occasionally molecular methods can be added.
